# Recognition and epileptology of protracted CLN3 disease

**DOI:** 10.1111/epi.17616

**Published:** 2023-04-27

**Authors:** Jillian M. Cameron, John A. Damiano, Bronwyn Grinton, Patrick W. Carney, Penny McKelvie, Peter Silbert, Nicholas Lawn, Ingrid E. Scheffer, Karen L. Oliver, Michael S. Hildebrand, Samuel F. Berkovic

**Affiliations:** ^1^ Department of Medicine, Epilepsy Research Centre University of Melbourne, Austin Health Melbourne Victoria Australia; ^2^ Eastern Health Clinical School Monash University Melbourne Victoria Australia; ^3^ Florey Institute of Neuroscience and Mental Health Heidelberg Victoria Australia; ^4^ Department of Pathology St Vincent's Hospital Melbourne Melbourne Victoria Australia; ^5^ Department of Medicine University of Western Australia Perth Western Australia Australia; ^6^ Royal Perth Hospital Perth Western Australia Australia; ^7^ Department of Paediatrics University of Melbourne Melbourne Victoria Australia; ^8^ Murdoch Children's Research Institute Parkville Victoria Australia; ^9^ Population Health and Immunity Division Walter and Eliza Hall Institute of Medical Research Parkville Victoria Australia; ^10^ Department of Medical Biology University of Melbourne Parkville Victoria Australia

**Keywords:** CLN3, epilepsy, neuronal ceroid lipofuscinosis

## Abstract

**Objective:**

This study was undertaken to analyze phenotypic features of a cohort of patients with protracted CLN3 disease to improve recognition of the disorder.

**Methods:**

We analyzed phenotypic data of 10 patients from six families with protracted CLN3 disease. Haplotype analysis was performed in three reportedly unrelated families.

**Results:**

Visual impairment was the initial symptom, with onset at 5–9 years, similar to classic CLN3 disease. Mean time from onset of visual impairment to seizures was 12 years (range = 6–41 years). Various seizure types were reported, most commonly generalized tonic–clonic seizures; focal seizures were present in four patients. Progressive myoclonus epilepsy was not seen. Interictal electroencephalogram revealed mild background slowing and 2.5–3.5‐Hz spontaneous generalized spike–wave discharges. Additional interictal focal epileptiform discharges were noted in some patients. Age at death for the three deceased patients was 31, 31, and 52 years. Molecular testing revealed five individuals were homozygous for c.461‐280_677 + 382del966, the "common 1‐kb" *CLN3* deletion. The remaining individuals were compound heterozygous for various combinations of recurrent pathogenic *CLN3* variants. Haplotype analysis demonstrated evidence of a common founder for the common 1‐kb deletion. Dating analysis suggested the deletion arose approximately 1500 years ago and thus did not represent cryptic familial relationship in this Australian cohort.

**Significance:**

We highlight the protracted phenotype of a disease generally associated with death in adolescence, which is a combined focal and generalized epilepsy syndrome with progressive neurological deterioration. The disorder should be suspected in an adolescent or adult patient presenting with generalized or focal seizures preceded by progressive visual loss. The common 1‐kb deletion has been typically associated with classic CLN3 disease, and the protracted phenotype has not previously been reported with this genotype. This suggests that modifying genetic factors may be important in determining this somewhat milder phenotype and identification of these factors should be the subject of future research.

**Key Points**

- Protracted CLN3 disease is easily missed and should be considered in an adolescent or adult patient presenting with generalized or focal seizures with prior visual failure
- Protracted CLN3 disease is best conceptualized as a combined focal and generalized epilepsy syndrome with progressive neurological deterioration
- Compared to classic CLN3 disease, seizure onset is typically much later in protracted CLN3 disease, and uniform early mortality is not seen
- No definitive genotype–phenotype correlation has emerged between classic and protracted forms of CLN3 disease

## 1 INTRODUCTION

Neuronal ceroid lipofuscinoses (NCLs) comprise a family of neurodegenerative diseases, associated with intracellular storage of abnormal lipopigment in neurons and peripheral tissues. CLN3 disease is one of the more common NCLs and is an autosomal recessive condition due to biallelic variants in *CLN3*.^1^ *CLN3* encodes a transmembrane protein that is predominantly located in the lysosome.^2^ The exact function of the CLN3 protein is unknown, but it is thought to have a role in multiple cellular processes including lysosomal pH homeostasis, vesicular trafficking, autophagy, and apoptosis.^3^

Classic CLN3 disease is characterized by childhood onset progressive severe visual loss due to retinal degeneration. Seizures typically occur within 2–4 years of the onset of visual disturbance. Progressive decline in motor function is seen, with the emergence of cerebellar, pyramidal, and extrapyramidal features in late childhood and early adolescence. Cognitive impairment occurs early in the disease course, with a precipitous decline to dementia over years.^4^ Overall, there is a rapidly progressive course, with death typically occurring prior to 20 years of age.^5^ Cardiac abnormalities, including conduction abnormalities and ventricular hypertrophy, and an autophagic vacuolar myopathy have been reported as associated systemic features.^6^, ^7^ Eighty percent of pathogenic alleles are an intragenic *CLN3* deletion c.461‐280_677 + 382del966, termed the "common 1‐kb deletion."^1^, ^8^

A rarer phenotype, known as protracted CLN3 disease, has been described more recently.^9^, ^10^, ^11^, ^12^ We identified 20 published cases of protracted CLN3 disease in the literature.^9^, ^10^, ^11^, ^12^, ^13^, ^14^, ^15^ Protracted CLN3 disease is also associated with childhood onset progressive visual loss; however, seizures and other neurological manifestations do not typically develop until the third decade of life or later. Although early mortality is still seen in this cohort, the life expectancy is longer than that of classic CLN3 disease. Clinically, we recognized that in the diagnostic evaluation of patients with protracted CLN3 disease, the relevance of an individual's visual impairment was not always appreciated, especially if the blindness had been incorrectly attributed to a primary retinal disorder. Here, we describe the clinical phenotypic features of 10 previously unreported patients from six families of protracted CLN3 disease and analyze genotype–phenotype correlations.

## 2 MATERIALS AND METHODS

### 2.1 Ascertainment

Five patients were ascertained through the Epilepsy Clinic at Austin Hospital, and five were referred via direct correspondence to the senior author. We systematically collated clinical data on age at disease onset, seizure types, neurological features, associated systemic features, cognition, and outcome. Onset of visual impairment was defined as the age at onset of clinically significant visual loss. Electrophysiological and neuroimaging data were collected where available and analyzed.

Five patients had a pre‐existing known diagnosis of protracted CLN3 disease identified by clinical molecular testing. In the other five patients, the diagnosis was suspected clinically, and confirmed on a research basis by targeted Sanger sequencing of the *CLN3* gene with or without prior whole exome sequencing. Segregation of the pathogenic variants was also determined by Sanger sequencing in each pedigree where DNA from additional family members was available.

The Austin Health Human Research Ethics Committee at Austin Hospital, Melbourne, Australia approved this study (Project No. H2007/02961). Written informed consent was obtained from all patients and relatives following local ethics committee requirements.

### 2.2 Relatedness and haplotype analysis

DNA was available for haplotype analysis from three recently ascertained families. One patient from each of three reportedly unrelated families (Individuals 1, 2A, and 4A) were genotyped for 725 875 single nucleotide polymorphisms (SNPs) using the Illumina Global Screening Array‐24. Relatedness between the three samples was estimated by identity by descent (IBD) analyses using KING^16^ and Tribes.^17^

Chromosome 16 SNP data were phased using Eagle2^18^ with the Michigan Imputation server (Minimac4).^19^ Haplotypes were reconstructed on either side of the *CLN3* locus and compared between cases using graphical and analytical methods with the statistical programming software R (version 4.1.1).

Haplotype age was estimated using an algorithm that predicts the age of the most recent common ancestor based on the lengths of shared regions utilizing recombination rates, available at https://shiny.wehi.edu.au/rafehi.h/mutation‐dating/.^20^

## 3 RESULTS

We identified 10 patients with protracted CLN3 disease from six reportedly unrelated families, including three sibling pairs and one pair of monozygotic twins. All were born in Australia and of White European background. There was no known consanguinity.

### 3.1 Disease onset and clinical course

In all patients, the first clinical feature was visual impairment beginning between 5 and 9 years of age (mean = 6.7 years). All patients experienced progressive severe visual deterioration. Six patients had previously received an alternative diagnosis regarding the underlying cause of their visual impairment, including Stargardt disease, cone‐rod dystrophy, and retinitis pigmentosa.

Seizures occurred in eight patients. The mean time from onset of visual impairment to onset of seizures was 12 years (range = 6–41 years). Ages of death for the three deceased patients were 31, 31, and 52 years. All other patients were between 19 and 49 years of age at last review.

### 3.2 Seizures and neurological features

A variety of seizure types were reported. Tonic–clonic seizures presumed to be generalized at onset were reported in seven patients. Myoclonic seizures occurred in two patients. Seizures with focal onset, including focal impaired awareness seizures, focal aware seizures, and focal to bilateral tonic–clonic seizures, were reported in four patients. Among those patients with focal seizures, clinical manifestations including oral automatisms and confusion were reported. Three of the four patients with reported focal seizures had focal interictal epileptiform discharges recorded on electroencephalogram (EEG; see Electroencephalography section below).

Seizure frequency was variable, with patients typically experiencing at least several seizures per year and variable response to antiseizure medications. Two patients never experienced any seizures. Progressive myoclonus epilepsy was not seen.

Cognitive impairment and cerebellar features including mild truncal ataxia and bilateral dysdiadochokinesis were the most common associated neurological abnormalities in the cohort. Progressive cognitive decline was present in nine patients. Remarkably, at time of last review, the remaining individual (Patient 2B) was engaged in full‐time administrative employment and functioning independently. Cervical dystonia was seen in one patient. No patients had features of an associated myopathic process.

### 3.3 Developmental history

Four patients (two sibling pairs) had developmental delay prior to the onset of visual impairment. One sibling pair (Patients 3A, 3B) had mild learning difficulties, delayed motor milestones, and normal speech. The other sibling pair (Patients 4A, 4B) had speech delay noted at 2 years of age and unremarkable motor development. The remainder of the cohort had an unremarkable developmental history.

### 3.4 Associated features

Cardiac abnormalities were seen in two patients, a sibling pair who both developed symptomatic bradycardia requiring insertion of a permanent pacemaker. One of these siblings also developed atrial flutter/fibrillation. Possible cardiac involvement was reported in a third patient, who experienced recurrent syncopal episodes, although cardiac investigations including Holter monitor and transthoracic echocardiogram were unremarkable.

### 3.5 Electroencephalography

**FIGURE 1:**
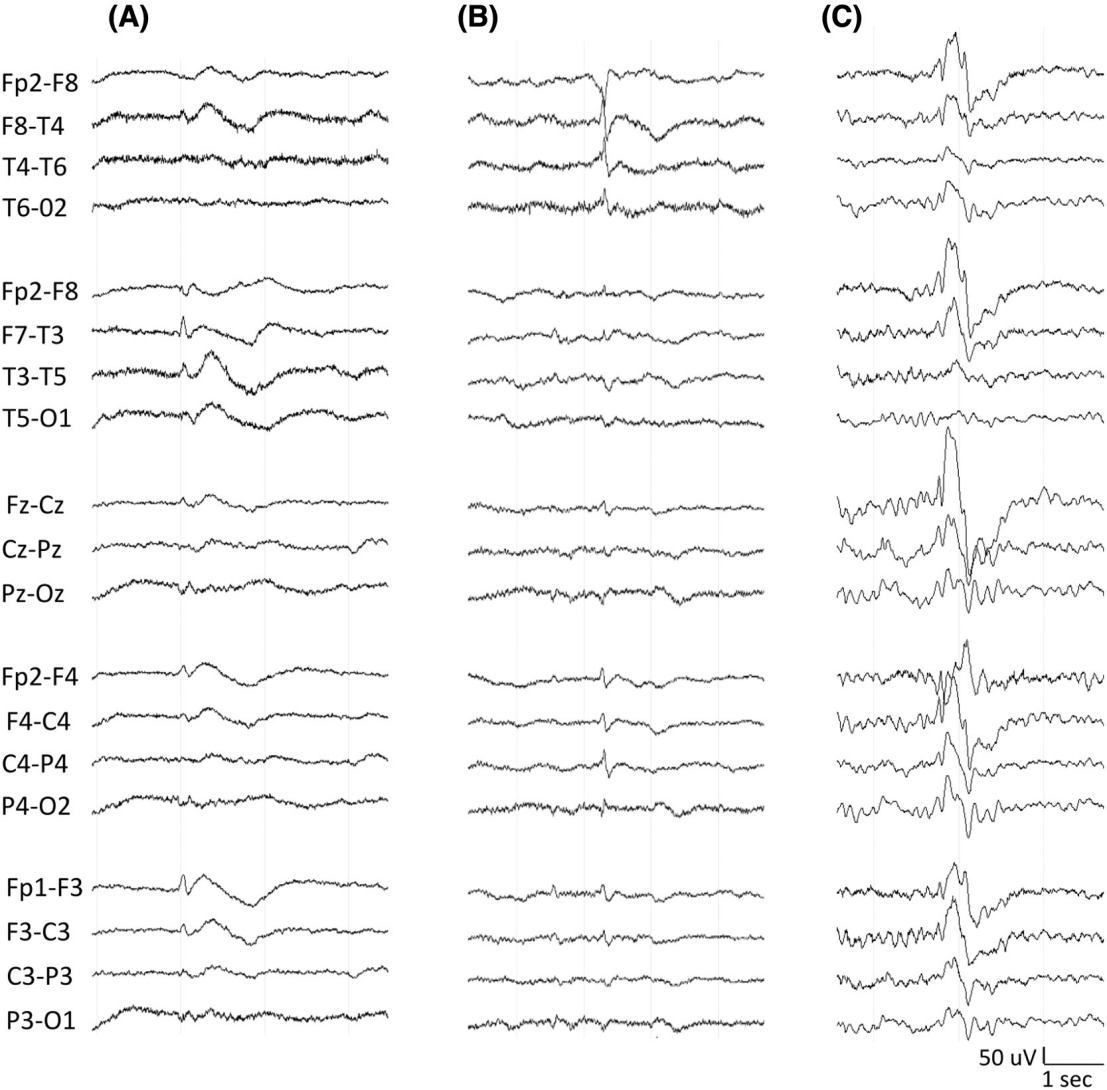
(A, B) Electroencephalograms (EEGs) from Patient 2A demonstrating independent bilateral frontotemporal sharp–slow discharges. (C) EEG from Patient 3A demonstrating generalized spike–wave discharge.

Electrophysiological data were available in six patients. EEG typically revealed mild background slowing and 2.5–3.5‐Hz generalized spike–wave discharges. Independent bilateral frontotemporal epileptiform discharges were seen in three patients (Figure 1). One patient had a photoparoxysmal response.

### 3.6 Neuroimaging

Magnetic resonance imaging (MRI) was conducted for eight patients. Age at MRI was between 6 and 50 years, and no patients had serial imaging. Four MRI brain images were available for independent expert neuroradiologist review. Mild–severe generalized cerebellar and mild–moderate cerebral atrophy was noted in all, with no other distinctive features seen.

### 3.7 Time to diagnosis

The median time from onset of visual impairment to confirmed genetic diagnosis was 12 years (range = 1 month–43 years). The patient with a molecular diagnosis 1 month after onset of visual impairment was a sibling of an affected patient. Among the patients with seizures, median time between first seizure and confirmed genetic diagnosis was 3 years (range = 2–9 years, excluding two patients with known genetic diagnosis prior to seizure onset). One patient was diagnosed postmortem after histopathological examination raised the possibility of an NCL.

### 3.8 *CLN3* genotypes

The six families harbored a combination of four recurrent pathogenic variants in *CLN3*. Five of six families carried at least one copy of the common 1‐kb deletion, and two families carried one copy of the p.Ser171Phe pathogenic variant. The p.G187Dfs*48 and p.Tyr389Asn pathogenic variants were unique to Siblings 3A and 3B (Table 1).

**TABLE 1:** Genotype and phenotypic features of individuals with protracted CLN3 disease.

ID	Gender	*CLN3* genotype	Age at visual impairment onset, years	Age at seizure onset, years	Age at last review, years	Seizures	Associated features
1	F	Common 1‐kb deletion (mat.), c.512C > T; p.Ser171Phe (pat.)	9	N/A	Deceased at age 52 years	Nil	Progressive cognitive decline, symptomatic bradycardia requiring PPM insertion
2A	F	Common 1‐kb deletion (mat.), c.512C > T; p.Ser171Phe (pat.)	6	47	49	FIAS, FAS	Progressive cognitive decline, mild bilateral dysdiadochokinesis, atrial fibrillation/flutter, symptomatic bradycardia requiring PPM insertion
2B	M		5	N/A	50	Nil	Ataxia, mild bilateral dysdiadochokinesis
3A	F	c.558_559delAG; p.G187Dfs*48 (mat.), c.1165 T > A; p.Tyr389Asn (pat.)	9	20	23	Frequent GTCS, myoclonus	Progressive cognitive decline, bilateral dysdiadochokinesis, recurrent syncopal episodes
3B	M		9	17	19	Rare GTCS	Progressive cognitive decline, mild truncal ataxia, bilateral dysdiadochokinesis
4A^a^	F	Homozygous common 1‐kb deletion	5	13	Deceased at age 31 years	Refractory GTCS	Progressive cognitive decline, ataxia
4B^a^	F		5	13	Deceased at age 31 years	Refractory GTCS	Progressive cognitive decline, ataxia
5A	M	Homozygous common 1‐kb deletion	6	12	21	Refractory GTCS, FIAS, FAS	Progressive cognitive decline, cervical dystonia
5B	M		6	12	23	Refractory GTCS, FIAS, FAS	Progressive cognitive decline, ataxia, dysphagia, behavioral disturbance
6	M	Homozygous common 1‐kb deletion	5	11	20	Frequent GTCS, FIAS, myoclonus	Progressive cognitive decline

*Note:* Patients with the same numeric identifier (e.g., 2A and 2B) are sibling pairs from the same family.

Abbreviations: F, female; FAS, focal aware seizures; FIAS, focal impaired awareness seizures; GTCS, generalized tonic–clonic seizures; M, male; mat., maternal allele; N/A, not available; pat., paternal allele; PPM, permanent pacemaker. ^a^Twin.

Although no genealogical relationship could be established between families that shared pathogenic variants, we sought to test this genetically in three families with available DNA. IBD analysis revealed an unexpected, close familial relationship (approximately second degree) between Patients 1 and 2A, who are both biallelic for the common 1‐kb deletion and p.Ser171Phe pathogenic variants. No close relationship was detected between Patient 6 and either Patient 1 or Patient 2A, despite all sharing at least one copy of the common 1‐kb deletion (up to seventh‐degree relatives excluded by Tribes).^17^

**FIGURE 2:**
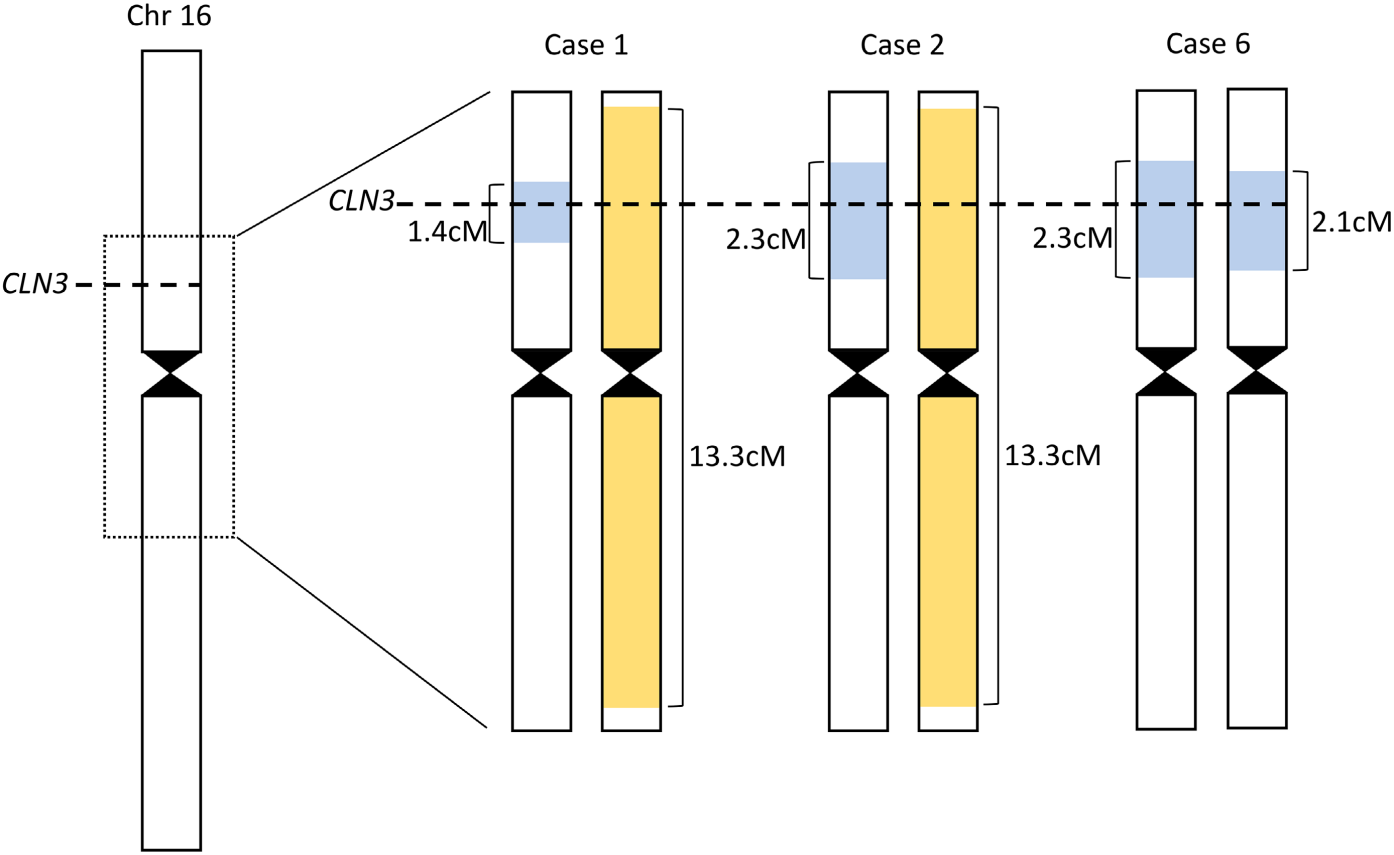
Schematic demonstrating common haplotype was observed for all three samples/families (Haplotype A in blue) and the common haplotype shared by Patients 1 and 2A (Haplotype B in yellow).

To further examine the relationship between Patients 1 and 2A, and to determine whether the common 1‐kb deletion may have been inherited from a distant common ancestor in all three families, the chromosome 16 haplotypes around the *CLN3* locus were examined (Figure 2). A common haplotype was observed for all three samples/families (Figure 2, Haplotype A). Consistent with the genotypes, Patient 4A was homozygous for the haplotype, whereas Patients 1 and 2A were heterozygous. The common region shared by all four alleles spanned 1.4 cM, with the longest shared region between any two alleles spanning 2.3 cM. Patients 1 and 2A also shared a second haplotype spanning approximately 13.3 cM (Figure 2, Haplotype B); this represents the p.Ser171Phe haplotype.

The age of Haplotype A was estimated to be 58.2 generations (95% confidence interval = 20.6–175.7 generations) or approximately 1450 years (95% confidence interval = 525–4400 years), assuming 25 years per generation.

## 4 DISCUSSION

This case series provides further insight into the rare condition of protracted CLN3 disease. In comparison to classic CLN3 disease, the onset of seizures in protracted CLN3 disease is much later, typically in adolescence or early adulthood, with visual failure occurring in childhood in both. Although early mortality is seen in the minority of patients with protracted CLN3 disease, overall survival is also more favorable, with survival seen into the fifth and sixth decades (Table 2).

**TABLE 2:** Comparison of key clinical and electrographic features of classic CLN3 disease and protracted CLN3 disease.

Feature	Classic CLN3 disease	Protracted CLN3 disease
Age at clinical onset, mean (range)	6 years (4–9)	6 years (5–9)
Symptom at clinical onset	Visual loss	Visual loss
Age at seizure onset, median (range)	10 years (5–16)	13 years (11–47)
Seizure disorder	Combined focal and generalized epilepsy	Combined focal and generalized epilepsy
EEG features	Background slowing GSW, focal, bilateral, multifocal interictal epileptiform discharges	Background slowing 2.5–3.5‐Hz GSW, independent bilateral frontotemporal interictal epileptiform discharges
Mortality	Death typically prior to 20 years of age	Variable early mortality reported in minority (30–50 years)
Developmental history	Typically unremarkable, although delayed motor and speech milestones seen in some	Typically unremarkable, although delayed motor and speech milestones seen in some
Cognition	Early onset rapidly progressive cognitive decline	Gradually progressive cognitive decline in majority
Associated features	Pyramidal, extrapyramidal, cerebellar features Cardiac conduction abnormalities Myopathy	Cerebellar features Cardiac conduction abnormalities

Abbreviations: EEG, electroencephalographic; GSW, Generalized Spike–Wave.

Previously, classic CLN3 disease was sometimes loosely considered along with CLN2 and CLN6 to have a progressive myoclonic epilepsy phenotype. More recent careful studies, however, show that myoclonus is not a predominant feature of classic CLN3 disease, and that the epilepsy type associated with classic CLN3 disease is best classified as a combined focal and generalized epilepsy.^21^ This case series highlights that the epilepsy type of protracted CLN3 disease is also a combined focal and generalized epilepsy. From a syndromic perspective, it has been suggested that classic CLN3 disease could be conceptualized as a developmental and epileptic encephalopathy (DEE).^21^ In protracted CLN3 disease, developmental milestones are generally within normal limits, although four of our cohort had a history of developmental issues affecting motor or speech domains, which preceded the emergence of visual loss. Progressive cognitive decline was noted in all but one patient. Using the new International League Against Epilepsy nomenclature, protracted CLN3 disease is an epilepsy syndrome with progressive neurologic deterioration, rather than a DEE.^22^

To date, no definitive genotype–phenotype correlation has emerged to explain the variable clinical manifestations between classic and protracted forms of the disease. To the contrary, to the best of our knowledge, this is the first description of protracted CLN3 disease phenotypes in patients homozygous for the common 1‐kb deletion. A trend toward earlier seizure onset in patients homozygous for the common 1‐kb deletion is noted; however, these individuals still present with a phenotype consistent with the protracted form of the disease.

From the available data, variant location does not seem to have a clear relationship with phenotype. CLN3 is a transmembrane protein, comprising six membrane‐spanning domains. Pathogenic missense variants associated with classic CLN3 disease are known to cluster at the luminal loops of CLN3 protein.^3^ The common 1‐kb deletion causes complete loss of exons 7 and 8, which form the second luminal loop, known to be the most highly conserved domain across species.^23^ This suggests that these luminal loops are critical sites for CLN3 function.^24^ Missense variants seen in our case series did not cluster in any particular area, mapping to luminal, cytosolic, and membrane locations on a topographical model.

Haplotype analysis provides evidence of a common founder for the 1‐kb *CLN3* deletion in at least four instances of the deletion in three families. Haplotype dating suggests the deletion arose approximately 1500 years ago in our sample, long predating White settlement of Australia.

This case series serves to highlight an adolescent and adult phenotype of a disease typically associated with adolescent mortality, raising awareness for adult epileptologists. In our cohort, time from initial clinical manifestation to genetic diagnosis was variable—in several patients, quite prolonged even after the onset of clinical seizures—suggesting that the clinical association with pre‐existing visual impairment may not be well recognized. Physician cognitive bias in the form of anchoring (being overly influenced by initial information—such as an apparent retinal syndrome diagnosis—with subsequent failure to modify the diagnosis on the basis of new information such as the development of seizures^25^) may impact diagnostic accuracy. Protracted CLN3 disease should be considered in an adolescent or adult patient presenting with generalized or focal seizures with prior visual failure, and in this context it would be appropriate to arrange molecular diagnostic testing to either confirm or exclude this diagnosis. In the era of more readily accessible molecular testing, the diagnostic role of skin biopsy is changing. Especially given variable local neuropathological expertise, biopsies are generally considered to provide additional confirmation when, for example, molecular diagnostics reveal variants of uncertain significance.

Accurate and timely diagnosis for these patients is important. On an individual level, it ends an often prolonged and frustrating diagnostic journey, providing insight into symptomatology and avoiding unnecessary additional investigations. It also facilitates identification and management of associated comorbidities, such as cardiac involvement in CLN3 disease. A genetic diagnosis has practical genetic counseling implications for family members and future planning. There are currently no approved disease‐modifying therapeutic options available for CLN3 disease; however, antisense oligonucleotides have recently been shown to improve survival in animal models of CLN3 disease,^26^ holding promise for future precision medicine therapeutic options.

## AUTHOR CONTRIBUTIONS

Jillian M. Cameron, John A. Damiano, Bronwyn Grinton, Karen L. Oliver, Michael S. Hildebrand, and Samuel F. Berkovic were involved in conception and design of the study, acquisition and analysis of data, and manuscript drafting and completion. All remaining authors were involved in acquisition and analysis of data and manuscript review.

## CONFLICT OF INTEREST STATEMENT

S.F.B. reports unrestricted educational grants from UCB Pharma, Chiesi, LivaNova, and Seer Medical and personal fees from Sequirus and Praxis during the conduct of this study. I.E.S. has served on scientific advisory boards for BioMarin, Chiesi, Eisai, Encoded Therapeutics, GlaxoSmithKline, Knopp Biosciences, Nutricia, Rogcon, Takeda Pharmaceuticals, UCB, and Xenon Pharmaceuticals; has received speaker honoraria from GlaxoSmithKline, UCB, BioMarin, Biocodex, Chiesi, LivaNova, and Eisai; and has received funding for travel from UCB, Biocodex, GlaxoSmithKline, BioMarin, and Eisai. None of the remaining authors has any conflict of interest to disclose. We confirm that we have read the Journal's position on issues involved in ethical publication and affirm that this report is consistent with those guidelines.
